# White bread as the final preexamination meal improves gastric cleanliness and visualization during magnetic capsule endoscopy

**DOI:** 10.1097/MD.0000000000048077

**Published:** 2026-03-20

**Authors:** Yan Song, Chao Huang, Hongxue Lu, Shanshan Hu, Jiancheng Zhang, Guanyu Zhou, Pu Wang

**Affiliations:** a Department of Gastroenterology, Sichuan Provincial People’s Hospital, School of Medicine, University of Electronic Science and Technology of China, Chengdu, China.

**Keywords:** gastric cleanliness, magnetically controlled capsule endoscopy, visualization, white bread

## Abstract

Suboptimal gastric preparation remains a critical factor limiting visualization efficacy in magnetically controlled capsule endoscopy (MCE). Notably, preliminary observations indicate that patients consuming white bread as their final pre-MCE diet showed improved gastric mucosal visibility. This study therefore seeks to evaluate the association between the final pre-procedural diet and gastric preparation quality in MCE examinations. A total of 230 patients scheduled for MCE were enrolled in this non-randomized controlled cohort study, with 115 patients received white bread as their pre-MCE diet the day preceding the examination, while patients in control group (n = 115) who consumed steamed or boiled rice was 1: 1 matched by age group and helicobacter pylori status. Gastric cleanliness and mucosal visualization quality were assessed by a panel of 3 independent endoscopists. Secondary outcome measures included comparative analysis of procedural duration and diagnostic yield for atrophic gastritis and polyps. The total score of gastric cleanliness in the white bread group was significantly higher than that in the control group, 22.45 versus15.85 (mean difference 6.63, 95% CI 6.05–7.16, *P* <.001). The total score of mucosal visualization in the white bread group was significantly better than that in the control group, 22.03 versus 13.44, (mean difference 8.59, 95% CI 7.94–9.24, *P* <.001). The control group had 6% of patients with inadequate cleanliness versus 0% in the white bread group, *P* = .021. The total examination duration of the white bread group was significantly shorter than that of the control group, 16.48min versus 27.76min (mean difference 11.28, 95% CI 9.75–15.33, *P* <.001). Consumption of white bread as pre-MCE diet the day preceding the examination was associated with significantly improved gastric cleanliness, as compared with steamed or boiled rice. Further research is needed on more standardized dietary protocols.

## 1. Introduction

Three-dimensional magnetic guidance enables precise navigation of capsule endoscopy, establishing magnetically controlled capsule endoscopy (MCE) as a noninvasive gastric examination method.^[1–5]^ Large-scaled studies have demonstrated the feasibility and safety^[5,6]^ of MCE and have shown that the accuracy of MCE in detecting focal lesions is comparable to that of conventional gastroscopy.^[6]^

Adequate gastric preparation is paramount for optimal visualization during MCE due to its inherent limitation in therapeutic intervention capabilities. Despite rigorous adherence to standardized pre-procedural fasting protocols and systematic administration of defoaming agents and mucolytic preparations, clinical observations reveal that a subset of patients persistently exhibit nonnegligible food chyme or fine debris in their gastric cavity that may significantly impair MCE visualization and lesion identification.^[7]^

Superior gastric cleansing efficacy in patients consuming Western-style pre-procedure dinners, predominantly white bread, has been observed, as compared to those receiving rice-based Chinese regimens, as steamed/boiled white rice. This may stem from different hydrolysis rates and effects on gastric emptying due to different dietary components.^[8]^

To investigate these potential associations, we designed a prospective cohort study aiming at comparing gastric preparation efficacy between 2 pre-procedural dietary groups: subjects consuming white bread versus those ingesting steamed/boiled white rice as their final diet prior to MCE. The primary endpoints included quantitative assessment of gastric cleanliness and mucosal visualization quality, with secondary analysis of procedural duration and diagnostic yield metrics for gastrointestinal lesions.

## 2. Materials and methods

### 2.1. Design

This study was a prospective, physician single-blinded, non-randomized cohort study. The study protocol was approved by the Institutional Reviewer Board of Sichuan provincial people’s hospital, No. 2023; No. 236, and informed consent was obtained from each enrolled patient before the MCE procedure. This study was registered as No. ChiCTR2300077780 at Chinese Clinical Trial Registry.

### 2.2. Patients’ enrollment

Consecutive patients, aged 18 to 90 years, referred for MCE in Sichuan provincial people’s hospital from June, 2023 to February, 2024 were eligible for enrollment. Patients with any of the following conditions were excluded: patients who had taken intestinal motility agents and digestive enzyme within 3 weeks before the MCE examination, patients who did not comply with dietary requirements, patients with dysphagia, active metal implants (such as cardiac pacemakers), history of gastrointestinal surgery, patients with diabetes and thyroid disease, pregnant women, patients who were unwilling to sign the informed consent.

### 2.3. *Grouping and intervention* (Fig. 1)

Eligible participants who consented to the study protocol were asked by the research assistant which pre-MCE diet they would like to accept without any suggestions or hint, 150 g standard toasted white bread (Metro AG manufactured, Chengdu), or 150 g steamed or boiled local white rice.

**Figure 1.**
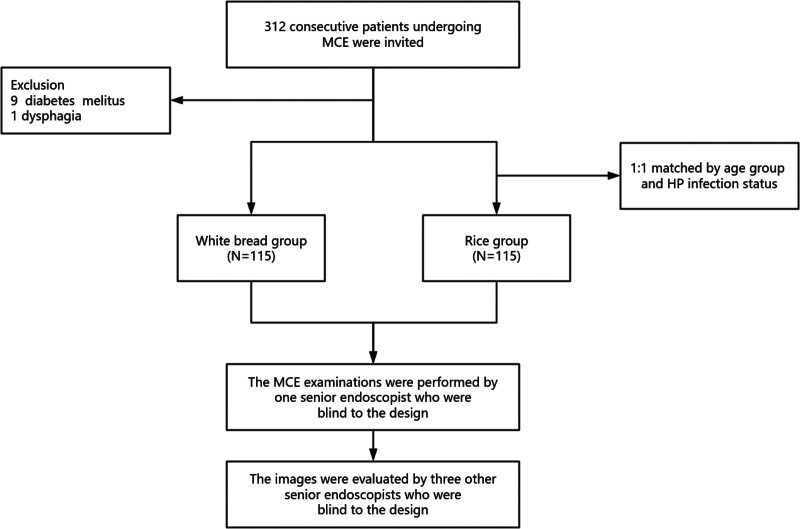
Schematic flow diagram of the study.

Participants selecting white bread comprised the study group. Rice consumers were matched with 1:1 ratio to the study group through age stratification (5-year intervals) and Helicobacter pylori (HP) infection status, establishing the control cohort.

The MCE capsule (Ankon Technologies Co. Ltd, Shanghai, China)^[6]^ can be controlled by using 2 joysticks with a guidance magnet robot. Each patient underwent examination according to standard operating procedures.^[7]^ After taking 750 mL pure water to fulfilling the stomach, patients were placed in a supine position. When the capsule entered the stomach, the examining physician operated the capsule endoscope to sequentially examine the cardia, fundus, body, angle, antrum and pylorus, twice each site. Images were captured at a speed of 6 frames per second. The examination time and any lesion being found were recorded. Three experienced endoscopists participated in the study as the examiners who were blinded to the study design.

### 2.4. Study outcomes

The primary outcome is gastric cleanliness and gastric mucosal visualization, while the secondary outcome is procedural duration and lesion detection rate which included atrophic gastritis and polypoid lesions.

A standard quality assessment scoring system of gastric preparation recommended by MCE guideline^[7]^ was used to evaluate the gastric cleanliness and gastric mucosal visualization. We meticulously documented the gastric cleanliness and mucosal visualization across 6 key anatomical regions of the stomach: the cardia, fundus, body, angulus, antrum, and pylorus. A guideline-recommended refined 4-point grading scale^[7]^ was employed to categorize gastric cleanliness, defining it as: excellent (score 4): no more than small bits of adherent mucus and foam, good (score 3): small amount of mucus and foam, but not enough to interfere with the examination, fair (score 2):considerable amount of mucus or foam present precluding a completely reliable examination and poor (score 1): large amount of mucus or foam residue.^[9–11]^ For evaluating mucosal visualization, a 4-point grading scale^[7]^ was utilized: Excellent (Score 4): over 90% of mucosa can be observed. Good (Score 3): Over 75% of the mucosa was observable. Fair (Score 2): over 50% of the mucosa was visible. Poor (Score 1): <50% of the mucosa was observable. Composite scores for overall gastric cleanliness and total mucosal visualization were derived by summing the individual scores across all 6 anatomical landmarks. This standardized evaluation framework enables systematic quantification of gastric preparation quality during MCE video analysis. Operationally, cases demonstrating cleanliness scores ≤2 across each anatomical segments were classified as unqualified cleanliness.

Three other experienced endoscopists blinded to study setting and patient allocation performed independent grade assessments of MCE-acquired images.

### 2.5. Statistical analysis

Due to the lack of any previous studies comparing the effects of these 2 diets on gastric cleanliness, we estimated the sample size through an internal exploratory pilot study. It was calculated that 115 patients for each group were needed with a power of 90% and a 2-sided significance level of .05 as well as a 10% more for possible withdrawal or drop out to obtain a 35% relative difference between groups.

Descriptive statistical methods were employed for data analysis. Categorical data were presented as frequencies and percentages, while continuous variables were described using means and standard deviations. For group comparisons, categorical data were analyzed using the Chi-square test or Fisher exact test as appropriate. The normality of continuous variables was assessed using the Shapiro–Wilk test. For data that followed a normal distribution, independent samples *t*-test was used for group comparisons; for non-normally distributed data, the Mann–Whitney *U* test was applied. All hypothesis tests were conducted with a significance level of α = 0.05. All statistical analyses were performed using R version 4.2.1.

## 3. Results

### 3.1. Patients baseline characteristics

230 patients were finally enrolled in this study, with 115 patients in study arm and another 115 matched patients in controlled arm. There were no statistically significant differences in demographic and major disease backgrounds between the 2 groups of patients, including age and gender ratio, body mass index, smoking history, alcohol consumption history, current infection of HP and indication for MCE (Table 1).

**Table 1:** Patients baseline characteristics.

	White bread group (n = 115)	Rice group (n = 115)	Mean difference/OR (95% CI)	*P*-value
Gender (male/female)	49/66	47/68	1.07 (0.65–1.74)	.789
Mean age (SD)	46.76 ± 10.89	49.56 ± 13.40	2.80 (−5.61 to 0.42)	.084
Mean BMI (SD)	23.61 ± 3.22	23.47 ± 3.58	0.14 (−0.80 to 0.79)	.769
Medical history				
Hypertension	8	11	1.59 (0.67–3.82)	.472
Smoking	16	17	0.73 (0.37–1.46)	.851
Alcohol drinking	12	11	0.95 (0.42–2.19)	.826
Helicobacter pylori infection	30	30	1.00 (1.00–1.00)	1.000
Indication				
Abdominal pain	16	28	0.49 (0.25–1.03)	.065
Abdominal distension	5	7	0.77 (0.23–2.57)	.767
Acid reflux or nausea or vomit	9	18	0.54 (0.24–1.24)	.101
Health examination	90	77	1.26 (0.98–1.63)	.076

BMI = body mass index, CI = confidence interval, SD = standard deviation.

### 3.2. Primary outcome

**Figure 2.**
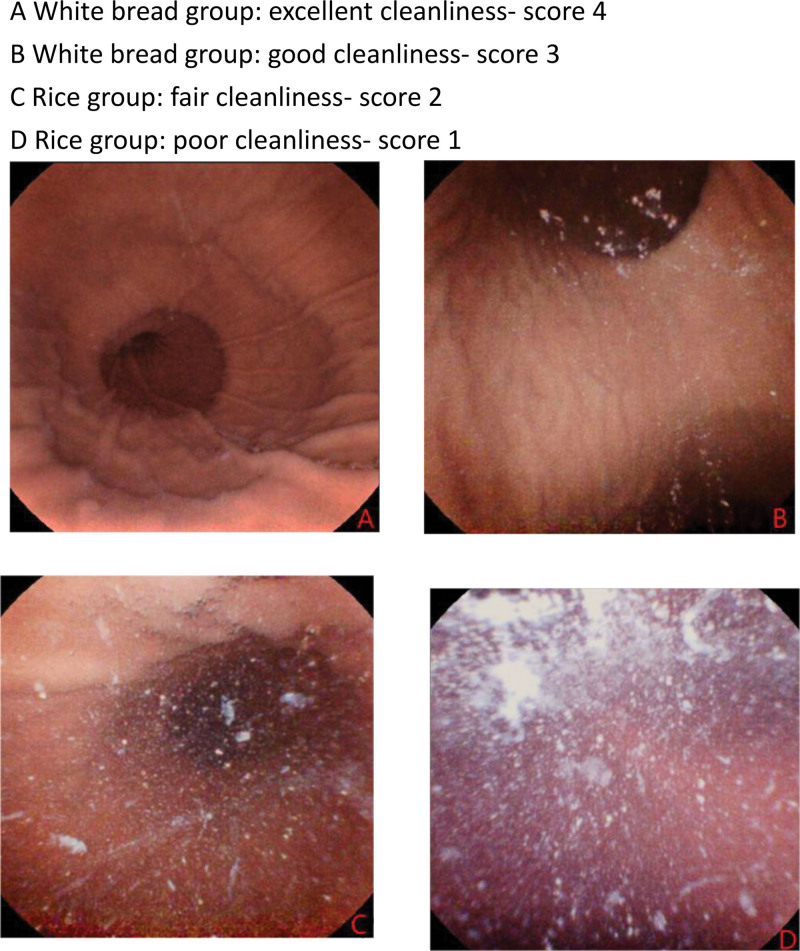
Gastric cleanliness of 2 groups. (A) White bread group: excellent cleanliness-score 4. (B) White bread group: good cleanliness-score 3. (C) Rice group: fair cleanliness- score 2. (D) Rice group: poor cleanliness-score 1.

Gastric preparation outcomes are detailed in Tables 2 and 3. The white bread group demonstrated superior gastric cleanliness 22.45 versus 15.85 (mean difference 6.63, 95% CI 6.05–7.16, *P* <.001) and mucosal visualization 22.03 versus 13.44 (mean difference 8.59, 95% CI 7.94–9.24, *P* <.001) compared to controls. All anatomical regions (cardia, fundus, body, angle, antrum, pylorus) exhibited significantly higher cleanliness and visualization scores in the intervention group. All anatomical regions in the white bread group achieved scores ≥3, approaching the maximum of 4 points (excellent). In controls, only antral/pyloric regions attained scores approximating 3 (good), while other regions averaged 2 (fair). Suboptimal preparation (any region <2 points) occurred in 6% (7/115) of controls versus 0% in the intervention group (*P* = .021, Fig. 2).

**Table 2:** Comparison of gastric cleanliness between white bread and rice.

Gastric region	Gastric cleanliness-score		Mean difference/OR (95% CI)	*P*-value
	White bread group	Rices group		
Cardia	3.75 ± 0.45	2.26 ± 0.72	1.51 (1.36–1.65)	<.001
Fundus	3.61 ± 0.50	2.21 ± 0.73	1.40 (1.25–1.56)	<.001
Body	3.40 ± 0.49	2.39 ± 0.63	1.01 (0.87–1.14)	<.001
Angulus	3.91 ± 0.28	2.95 ± 0.45	0.95 (0.85–1.04)	<.001
Antrum	3.77 ± 0.42	3.00 ± 0.54	0.77 (0.63–0.87)	<.001
Pylorus	3.98 ± 0.13	3.09 ± 0.49	0.88 (0.80–0.97)	<.001
Total score	22.45 ± 1.02	15.82 ± 3.12	6.63 (6.05–7.16)	<.001

CI = confidence interval.

**Table 3:** Comparison of gastric mucosal visualization between white bread and rice.

Gastric region	Gastric mucosal visualization score		Mean difference (95% CI)	*P*-value
	White bread group	Rice group		
Cardia	3.60 ± 0.60	2.34 ± 0.94	1.26 (1.04–1.48)	<.001
Fundus	3.45 ± 0.61	2.23 ± 0.96	1.22 (1.00–1.44)	<.001
Body	3.35 ± 0.61	2.24 ± 0.86	1.11 (0.90–1.32)	<.001
Angulus	3.91 ± 0.28	2.92 ± 0.55	0.99 (0.87–1.12)	<.001
Antrum	3.80 ± 0.40	2.92 ± 0.70	0.88 (0.73–1.03)	<.001
Pylorus	3.91 ± 0.28	3.12 ± 0.56	0.79 (0.67–0.91)	<.001
Total score	22.03 ± 2.32	13.44 ± 3.22	8.59 (7.94–9.24)	<.001

CI = confidence interval.

### 3.3. Secondary outcome

Procedural duration was significantly reduced in the white bread group compared to controls16.48 minutes versus 27.76 minutes (mean difference 11.28, 95% CI 9.75–15.33, *P* <.001) (Table 4).

**Table 4:** Procedural duration and lesion detection.

	White bread group (n = 115)	Rice group (n = 115)	Mean difference (95% CI)	*P*-value
Procedural duration	16.48 ± 4.19	27.76 ± 10.21	11.28 (9.75–15.33)	<.001
Lesion detection				
Chronic atrophic gastritis	42	42	1.00 (1.00–1.00)	1.000
Gastric polyps	10	8	0.79 (0.29–2.07)	.623

CI = confidence interval.

There was no significant difference in the detection rate of major gastric diseases such as chronic atrophic gastritis and gastric polyps between the 2 groups (Table 4).

## 4. Discussion

Mucosal obscuration by mucus, bubbles, and food debris constitutes primary impediments to comprehensive gastric mucosal inspection.^[12,13]^ This necessitates meticulous preprocedural preparation for MCE, given its inability for intraluminal re-cleansing in cases of suboptimal preparation.^[14]^ Preprocedural administration of mucolytic/defoaming agents (e.g., pronase, simethicone) and sodium bicarbonate has demonstrated efficacy in reducing gastric mucus and bubble formation, thereby enhancing mucosal visualization.^[15–17]^ However, these regimens demonstrate limited efficacy against chyme and fine particulate food residues, which persistently compromise mucosal visualization. Dietary selection emerges as a critical modifiable factor influencing residual gastric content. Notably, current evidence regarding dietary optimization protocols for MCE preparation remains sparse. This study may represent the first controlled evaluation of pre-procedural dietary protocols, motivated by empirical observations: patients consuming white bread as their final pre-MCE meal exhibited superior gastric cleanliness compared to those ingesting steamed or boiled white rice.

Dietary impacts on gastric preparation arise from 2 key factors: gastric emptying rate and food decomposition dynamics. Slowed gastric emptying induced by specific foods increases risk of chyme retention during examination. Concurrently, delayed intragastric breakdown of dietary components leads to persistent particulate residues, even after bulk food evacuation. These dual mechanisms critically determine mucosal visualization quality.

Gastric emptying studies demonstrate accelerated emptying rates following white bread consumption compared to rice and noodles,^[18]^ aligning with our clinical MCE observations. Notably, gluten content inversely correlates with gastric emptying rate – whole wheat bread (high gluten) exhibits slower emptying rate than white bread (low gluten),^[19,20]^ justifying our selection of optimal white bread variants. Regarding hydrolysis dynamics, white bread achieves 90% gastric starch hydrolysis within 40 minutes,^[21]^ contrasting significantly with rice’s 20% hydrolysis at 40 minutes and 80% at 180 minutes.^[22]^ This superior hydrolysis kinetics contributes to reduced residual particulate burden.

White bread and rice share complex nutritional profiles as carbohydrate-rich foods, It is still unknown which specific components play the most important role, which is worthy of further study. For example, in the known knowledge, white bread contains more resistant starch (RS), especially its subtype RS3 than short- to medium-grain white rice.^[23,24]^ RS3 not only promotes gastrointestinal motility, shortening intestinal transit time, increasing the volume of defecation but also hydrolyzes more quickly,^[9,25–27]^ and thus may be one of the beneficial ingredients.^[28,29]^ The impact of specific components inside food is worthy of further investigation.

Moreover, prior studies indicate poorer proximal gastric preparation quality, potentially due to reduced cleansing agent contact from mucosa caused by gastric motility patterns and residual food sedimentation in left lateral positioning.^[4]^ Consistent with this, the rice group attained adequate preparation (score ≥3) only in antral/pyloric regions, while proximal segments averaged >2 (fair). The white bread group maintained consistent high scores (3.5–4) across all regions, suggesting its enhanced gastric emptying and rapid hydrolysis properties overcome regional preparation challenges. Superior preparation quality directly correlated with shorter procedure duration, potentially improving patient compliance and procedural safety.

This study has several limitations. First, the sample size calculation prioritized gastric cleanliness metrics over lesion detection power, potentially underpowering comparative analyses of the diagnostic yields, however, a cleaner stomach cavity is always more likely to lead to an increase in detection rate, which can be revealed through long-term larger-scale cohort studies. Second, dietary protocol lacked precision dosing adjustments for individual parameters influencing gastrointestinal physiology (e.g., body mass, metabolic status), and rice varieties were not standardized, limiting nutritional generalizability. Third, the lack of randomization in patient assignment introduces the potential for selection bias, despite age and HP status matching. Fourth, residual confounding from unmeasured variables affecting gastrointestinal motility remains possible. Finally, single-center recruitment from a rice-consuming Chinese population constrains external validity across diverse dietary cultures, People from different regions may not have the same reaction to the same food, so specialized research from different regions is needed to verify the conclusions of this study.

In conclusion, white bread has been found to be associated with improved gastric cleanliness and mucosal visualization during MCE examinations relative to rice-based preparations when consumed as the final pre-MCE diet. These findings underscore the necessity for protocol standardization and multi-center validation studies to optimize the pre-MCE dietary preparation.

## Acknowledgments

We thank Dr. Hao Yang from Statistical Department for providing insightful suggestions and help on the statistical analysis.

## Author contributions

**Conceptualization:** Yan Song, Guanyu Zhou, Pu Wang.

**Data curation:** Yan Song, Pu Wang.

**Formal analysis:** Pu Wang.

**Funding acquisition:** Pu Wang.

**Investigation:** Yan Song, Chao Huang, Hongxue Lu, Shanshan Hu, Jiancheng Zhang.

**Methodology:** Yan Song, Pu Wang.

**Resources:** Pu Wang.

**Supervision:** Pu Wang.

**Writing – original draft:** Chao Huang, Guanyu Zhou, Pu Wang.

**Writing – review & editing:** Pu Wang.
